# Obesity in adolescents associated with vascular aging – a study using ultra-high-resolution ultrasound

**DOI:** 10.48101/ujms.v127.8676

**Published:** 2022-06-03

**Authors:** Tord Naessen, Peter Bergsten, Tobias Lundmark, Anders Forslund

**Affiliations:** a Department of Women’s and Children’s Health, Uppsala University, Uppsala, Sweden; b Department of Medical Cell Biology, Uppsala University, Uppsala, Sweden

**Keywords:** Adolescent, obesity, inflammation, vascular aging, subclinical atherosclerosis, ultra-high-resolution ultrasound (UHRU), radial artery, intima thickness, intima/media thickness ratio, carotid IMT

## Abstract

**Background:**

Obesity in adolescents is increasing worldwide and associated with an elevated cardiovascular risk later in life. In a group-comparative study, we investigated the association between adiposity in adolescents and signs of vascular aging and inflammation.

**Methods:**

Thirty-nine adolescents (10–18 years old), 19 with obesity and 20 with normal weight, were enrolled. The intima thickness and intima/media thickness ratio (I/M) were assessed using high-resolution ultrasound in the common carotid artery (center frequency 22 MHz) and the distal radial artery (RA; 50 MHz). Increased intima and high I/M are signs of vascular aging. Body characteristics, high-sensitivity C-reactive protein (hs-CRP), plasma lipids, and glycemic parameters were measured.

**Results:**

Adolescents with obesity, compared to normal-weight peers, had elevated plasma lipid, insulin c-peptide, and hs-CRP levels, the latter increasing exponentially with increasing adiposity. Obese adolescents had a thicker RA intima layer [0.005 mm; 95% confidence intervals (0.000, 0.009); *P* = 0.043] and a higher RA I/M [0.10; (0.040, 0.147); *P <* 0.0007]. Group differences for the RA I/M remained significant after adjustment for age, sex, fasting plasma insulin, and body mass index, both separately and together (*P =* 0.032). The RA I/M was correlated with hs-CRP, and both were correlated with the analyzed cardiovascular risk factors. Receiver operating curve c-values for RA I/M (0.86) and hs-CRP (0.90) strongly indicated correct placement in the obese or non-obese group.

**Conclusions:**

Adolescents with obesity had significantly more extensive vascular aging in the muscular RA, than normal-weight peers. The findings support an inflammatory link between obesity and vascular aging in adolescents.

## Introduction

Obesity is increasing worldwide, more so in adolescents than in adults (1). Severe adiposity in adolescents is associated with worse cardiac and vascular structures and functions, (2) and child and adolescent obesity are associated with an increased risk of cardiovascular disease (CVD) events later in life (3). In American children with obesity who were killed in motor accidents (aged 10–14 years), over 50% showed signs of early atherosclerosis (4).

Thickening of the arterial intima layer is an early morphological sign of developing atherosclerosis, an inflammatory process that is the underlying cause of CVD (5). An increase in C-reactive protein (CRP) levels, measured using a high-sensitivity CRP (hs-CRP) test, is associated with obesity (2) and is correlated with early progression of carotid atherosclerotic activity (6). Conventional measurements of the common carotid intima-media thickness (CIMT) are sometimes, but not always, associated with measurements of body mass index (BMI), waist circumference, diastolic blood pressure (DBP), and hs-CRP in children and adolescents (7). However, because 8–12 MHz probe frequencies in conventional CIMT measurement are unable to assess the intima and media (muscle layer) thicknesses separately, CIMT does not reflect the differential changes in these layers during vascular aging/atherosclerosis development, i.e. the increased arterial intima thickness and, in the long run, decreased media thickness (8, 9). Furthermore, CIMT is not recommended for risk assessment of first atherosclerotic CVD events, according to recent guidelines (10). Since many years, we measure the intima thickness and the intima/media thickness ratio (I/M) to reflect vascular aging and subclinical atherosclerosis (11–14).

The aim of this study was to investigate differences between adolescents with and without obesity with regard to signs of vascular aging, as assessed by ultra-high-resolution ultrasound (UHRU), and to test associations with hs-CRP levels and variables reflecting adiposity and cardiovascular risk.

## Patients and methods

This study was performed at a university research clinic in Uppsala, Sweden, as part of the Uppsala Longitudinal Study of Childhood Obesity (15). We evaluated 39 adolescents (14 females and 25 males) aged between 10 and 18 years. Inclusion criteria were a standardized (age- and gender-adjusted) BMI (s-BMI) of >30 for subjects with obesity and a lean s-BMI for subjects of normal weight. Adolescents of normal weight were recruited by advertisement in the local media and from local schools, as previously described (15). Exclusion criteria were as follows: congenital syndromes, type-1 diabetes, hereditary hypercholesterolemia, homocysteinurinemia, secondary hypertension, antihypertensive therapy, chronic kidney disease, and coarctatio aortae. For controls, additional exclusions were diabetes mellitus I and II and medications affecting the blood pressure. The overwhelming majority of participants in both study groups were Caucasians; we had no information for one adolescent in the control group and three in the obese group. After receiving oral and written information on the study, all participants signed an informed consent form prior to inclusion. This study was approved by the Ethics Committee, Uppsala University, Uppsala, Sweden (Dnr. 2012/318).

### Anthropometric measurements

Height was measured to the nearest 0.1 cm, using a digital instrument (Busse, Ulmer Stadiometer, Elchingen, Germany), with the participant standing against the wall without shoes. We used the mean of two assessments. Weight was assessed in light clothing, but without shoes, on a digital weighing scale (SECA 704, Hamburg, Germany); the weight was reduced by 0.5 kg to allow for clothing. BMI was calculated as weight (kg)/height (m)^2^. The waist (between lowest costae and iliac crest), and the middle of the neck, upper arm, and upper thigh circumferences were measured to the nearest mm. The sagittal abdominal diameter (SAD) was measured to the nearest mm using an Abdometer (Novo Nordisk, Copenhagen, Denmark), with the subject lying on a flat horizontal hard bed with knees bent, during a normal expiration. Skinfold thicknesses were measured to the nearest 0.2 mm in the standing position (by the same technician for all participants) at biceps, triceps, subscapularis, and suprailiac measuring sites, using a Harpenden Skinfold Caliper (Baty International, Burgess Hill, West Sussex, UK) (15). The mean of two assessments was used in the analysis.

### Blood pressure

The same research nurse (MD) measured the BP of all participants on the right upper arm after they had rested for 15 min in the supine position, using a CAS 740 (CAS Medical Systems, Inc, Branford, CT, USA), cuff-size 12 × 35 cm or as appropriate. The mean of two measurements, 1 min apart, was used in the analysis.

### High-resolution ultrasound of the common carotid artery

The right common carotid artery (CCA) wall layers were imaged using high-resolution ultrasound fitted with a broad-band probe with a 22 MHz center frequency (Collagenoson^®^, Minhorst Company, Meudt, Germany), as extensively described previously (11, 14). Analysis was made off-line, and means of about 10 technically acceptable measurements were calculated and used in the analysis. The coefficient of variation (CV) was about 3.9% for the CCA intima thickness and about 3.4% for the media thickness (13).

### Ultra-high-resolution ultrasound of the distal radial artery

The left distal radial artery (RA) was imaged, about 1–2 cm proximal to the styloid process, using a Vevo 2100 ultrasound machine (Fujifilm VisualSonics Inc., Toronto, Canada) fitted with a 50 MHz peak frequency linear transducer, applied without pressure and as previously described (16). Estimates from the M Mode were used because they were closer to reported histomorphometry results than estimates from the B Mode. In case of intima thickening, presented as a double-lined structure, the thickness was measured from leading–leading edge (Supplemental Figure 1). All measurements of scans were performed off-line and blindly with regard to study subject characteristics and by the same researcher (TN). The mean of about three technically acceptable assessments was used as the final estimate of the layer dimensions. The CV was 7% for the RA intima thickness and 4% for the media thickness (16).

### Blood sampling and plasma analysis

Blood was sampled as previously described (15). In short, blood was drawn from a peripheral vein using a stationary catheter. After sampling, it was added to ethylenediaminetetraacetic acid tubes, centrifuged at 4°C, and the plasma was stored at −72°C until analyzed. Analyses of glucose (Abbott Architect, Abbott Diagnostics, Lake Forest, IL), insulin (Cobas E602, Roche Diagnostics, Indianapolis, IN), hs-CRP, and whole blood HbA1c were conducted at the Uppsala University Hospital laboratory, in accordance with local standard operating procedures. Insulin analyses were not carried out on blood samples with hemolysis.

### Oral glucose tolerance test

Oral glucose tolerance tests were conducted as previously described (17). In brief, the subject drank a glucose solution (1.75 g glucose per kg body mass, maximum 75 g), and blood samples were collected at –5, 5, 10, 30, 60, 90, and 120 min.

### Statistical analysis

Data are presented as medians with the interquartile range (25–75%). Comparisons between the groups were assessed using the Mann–Whitney U test, and comparisons of medians with 95% confidence intervals (CI) were assessed using the Hodges–Lehmann estimator. Correlations between variables were assessed using the Spearman rank correlation test. Non-parametric partial regression was used to adjust for covariates. Receiver operating characteristic (ROC) curve analysis was undertaken to illustrate the ability of RA I/M thickness ratio and hs-CRP values to correctly indicate the group to which the individual belonged. JMP version 15.0 (SAS Institute Inc., Cary, NC) and SPSS version 27 software packages were used. All tests were two-sided, and *P <* 0.05 was considered statistically significant.

## Results

**Figure 1:**
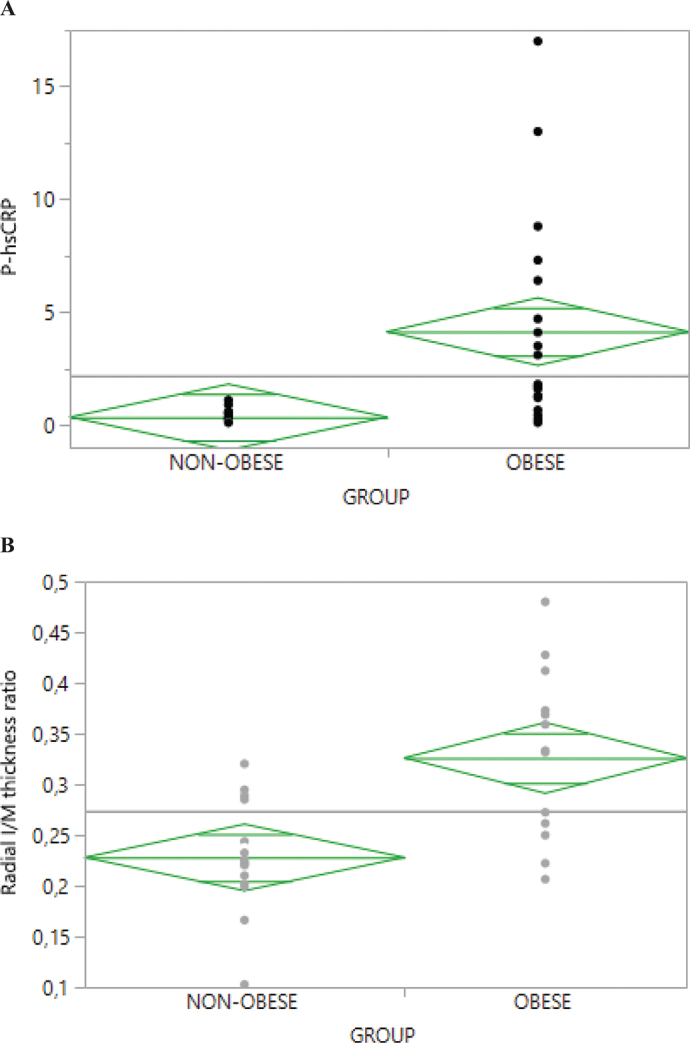
Plasma high-sensitivity C-reactive protein (hs-CRP) levels (**A**) and radial artery intima/media thickness ratios (**B**) in adolescents with and without obesity. P < 0.001.

Adolescents with obesity had significantly higher median values for weight, BMI, all parameters regarding body fat distribution, skinfold thicknesses, and systolic blood pressure (SBP) than adolescents of normal weight. Furthermore, obese adolescents had significantly higher plasma hs-CRP levels with a considerably wider range (Figure 1A), higher HbA1c, fasting plasma insulin, proinsulin, and c-peptide levels, and more adversely affected serum lipid and apolipoprotein profiles (Table 1).

**Table 1:** Clinical characteristics that differed significantly between study groups.

Variable	Obese adolescents (*n* = 19)	Normal-weight adolescents (*n* = 20)
	Median (IQR)	Median (IQR)
Weight (kg)	83 (78, 107)^c^	48 (44, 62)
BMI (kg/m^2^)	32 (30, 36)^c^	19 (18, 21)
SBP (mmHg)	115 (110, 128)^c^	106 (103, 109)
Plasma hs-CRP (mg/L)	1.8 (1.2, 6.4)^c^	0.26 (0.20, 0.47)
**Radial artery (*N* = 32)**	(*n* = 15)	(*n* = 17)
Intima thickness (mm)	0.028 (0.021, 0.032)^a^	0.023 (0.019, 0.026)
Intima/media ratio	0.333 (0.261, 0.373)^c,d^	0.223 (0.202, 0.265)
**Plasma lipids**		
LDL cholesterol (mmol/L)	2.6 (2.4, 3.2)^a^	2.3 (1.9, 2.7)
HDL cholesterol (mmol/L)	1.0 (0.9, 1.2)^c^	1.4 (1.2, 1.5)
LDL/HDL ratio	2.8 (2.1, 3.1)^c^	1.6 (1.4, 2.2)
Triglycerides (mmol/L)	1.1 (0.9, 1.4)^b^	0.7 (0.5, 0.8)
Apo A1 (g/L)	1.2 (1.1, 1.8)^a^	1.3 (1.2, 1.5)
Apo B (g/L)	0.8 (0.7, 0.9)^b^	0.6 (0.5, 0.7)
Apo B/A1	0.6 (0.5, 0.8)^b^	0.5 (0.4, 0.6)
**Glycemic parameters**		
HbA1c (whole blood)	34 (32, 36)^b^	32 (30.3, 33)
Fasting		
Insulin	23 (13, 34)^c^	7.5 (5.3, 10.8)
Proinsulin	18.4 (9.63, 35)^c^	7.1 (5.1, 8.8)
C-peptide	1.2 (0.86, 1.4)^c^	0.54 (0.41, 0.54)
**Bodyfat distribution**		
BMI SDS	2.9 (2.6, 3.3)^c^	–0.1 (–1, 0.7)
SAD	25 (22, 27)^c^	17 (15, 19)
SAD/height	0.15 (0.14, 0.16)^c^	0.10 (0.09, 0.11)
Waist circ. (cm)	100 (94, 116)^c^	68 (63, 75)
Waist circ./height ratio	0.62 (0.58, 0.65)^c^	0.43 (0.40, 0.44)
Waist/hip ratio	0.92 (0.89, 0.94)^c^	0.82 (0.78, 0.84)
Upper thigh circ. (cm)	61 (56, 65)^c^	45 (43, 50)
Neck circ. (cm)	38 (35, 42)^c^	32 (30, 34)
Upper arm circ. (cm)	34 (32, 40)^c^	25 (24, 28)
**Skinfold measurements**		
Biceps (mm)	18.4 (14.3, 19.4)^c^	5.1 (4.2, 7,0)
Triceps (mm)	30.9 (28.3, 36.7)^c^	10.3 (8.5, 13.9)
Subscapular (mm)	32.4 (23.5, 37.6)^c^	7.4 (6.1, 9.4)
Supra-iliac (mm)	36 (26.3, 44.5)^c^	8.2 (6.1, 11.3)

^a^ *P* < 0.05, ^b^ *P* < 0.01, ^c^ *P* < 0.001, and ^d^ *P* = 0.031 after adjustment for age, sex, fasting plasma insulin, and BMI, respectively.

Data are presented as medians (25% and 75% IQR). The Wilcoxon sum rank test was used to test group differences.

Abbreviations: IQR, interquartile range; BMI, body mass index; SBP, systolic blood pressure; circ., circumference; hs-CRP, high-sensitivity C-reactive protein; LDL, low-density lipoprotein; HDL, high-density lipoprotein; SDS, standard deviation score; SAD, sagittal abdominal diameter.

### Signs of vascular aging

Adolescents with obesity had a significantly thicker RA intima layer [0.005 mm; 95% CI (0.000, 0.009); 0.043], and a significantly higher RA I/M [0.10; 95% CI (0.040, 0.147); *P* = 0.0007] than their non-obese peers (Table 1 and Figure 1B). The group differences for RA I/M remained significant after adjustment for age, sex, fasting plasma insulin, and BMI, both separately and together (*P <* 0.031), but not after adjustment for SAD/height. The RA wall thickness could not be assessed in four obese and three non-obese adolescents for logistical reasons. No significant group differences were found for the CCA wall dimensions. For clinical characteristics by group, lacking statistical significance, see Supplemental Table 1.

### Test of correlations

Most of the correlation tests showed stronger correlations between hs-CRP levels and CVD risk factors than between RA I/M and CVD risk factors. hs-CRP levels were significantly correlated with most of the serum lipid and apolipoprotein profiles analyzed, whereas both RA I/M and hs-CRP results were correlated with all body fat and skinfold measurements analyzed and with several glycemic variables (Table 2). The hs-CRP levels were correlated with SBP and appeared to increase logarithmically with increasing adiposity, with a significant quadratic fit for e.g. the triceps skinfold (*t* ratio = 3.87; *P <* 0.001) and the waist/height ratio (*t* ratio = 2.84; *P <* 0.01) (Supplemental Figure 2A–B). The RA I/M results were correlated with all body characteristic and skin folds, except neck circumference; and correlated most strongly with SAD/height (Spearman rank correlation coefficient, *r*_s_ = 0.62; *P* < 0.001) (Supplemental Figure 2C) but correlated also with hs-CRP (*r*_s_ = 0.38; *P =* 0.033) (Table 2). Of the anthropometric variables tested, SAD/height showed the strongest correlation with signs of subclinical atherosclerosis, with RA intima thickness (*r*_s_ = 0.50; *<*0.005, not shown) as well as with radial I/M ratio (0.62; <0.001) (Table 2). Among the measurements of waist circumference, HbA1c, fasting insulin, and C-peptide, the waist circumference had the strongest correlation with the RA intima thickness (*r*_s_ = 0.51; *P =* 0.009) (not shown), while the glycemic parameters correlated more strongly with RA I/M (fasting insulin: *r*_s_ = 0.57, *P <* 0.001; c-peptide: *r*_s_ = 0.52, *P =* 0.003) (Table 2). Associations with the combined intima-media thickness (IMT) in both the carotid and radial arteries were few, were often inconsistent, and were not significant. Correlations between the artery wall dimensions of the CCA and those of the RA were weak and not significant; the correlation between the RA intima thickness and the CIMT was numerically strongest (*r*_s_ = 0.33; *P =* 0.07) (not shown).

**Table 2:** Significant associations within the combined study groups of adolescents with or without obesity.

Variable	BMI	f-Insulin	hs-CRP	RA-I/M ratio	Systolic BP	LDL/HDL
	*r*_s_	*r*_s_	*r*_s_	*r*_s_	*r*_s_	*r*_s_
BMI (kg/m^2^)	1	0.73^c^	0.72^c^	0.49^b^	0.60^c^	0.53^b^
Waist-hip ratio	0.80^c^	0.64^c^	0.59^c^	0.47^b^	0.52^c^	0.56^c^
SBP (mmHg)	0.60^c^	0.57^c^	0.48^b^	0.18	1	0.26
DBP (mmHg)	0.24	0.39^a^	0.25	0.20	0.72^c^	0.20
P hs-CRP (mg/L)	0.72^c^	0.61^c^	1	0.38^a^	0.48^b^	0.47^b^
**Radial artery (*N* = 32)**						
Intima thickness	0.39^a^	0.42^a^	0.28	0.65^c^	0.21	-0.00
Media thickness	-0.02	-0.18	-0.15	-0.48^b^	0.20	-0.26
Intima/media ratio	0.49^b^	0.57^c^	0.38^a^	1	0.18	0.19
**Plasma lipids**						
Total cholesterol (mmol/L)	0.07	0.16	0.21	0.26	-0.05	0.45^b^
LDL cholesterol (mmol/L)	0.34^a^	0.30	0.41^a^	0.27	0.02	0.80^c^
HDL cholesterol (mmol/L)	-0.61^c^	-0.45^b^	-0.42^b^	-0.02	-0.40^a^	-0.83^c^
LDL/HDL ratio	0.53^b^	0.48^b^	0.47^b^	0.19	0.26	1
Triglycerides (mmol/L)	0.62^c^	0.65^c^	0.49^b^	0.21	0.51b	0.72^c^
Apo A1 (g/L)	-0.29	-0.16	-0.11	0.23	-0.20	-0.70^c^
Apo B (g/L)	0.51^c^	0.48^b^	0.55^c^	0.32	0.16	0.82^c^
Apo B/A1	0.47^b^	0.42^b^	0.46^b^	0.16	0.14	0.94^c^
**Glycemic parameters**						
HbA1c (whole blood)	0.47^b^	0.55^c^	0.29	0.32	0.21	0.47^b^
f-Insulin	0.73^c^	1	0.61^c^	0.57^c^	0.57^c^	0.48^b^
f-Proinsulin	0.70^c^	0.82^c^	0.59^c^	0.31	0.53^b^	0.51^b^
f-C-peptide	0.79^c^	0.95^c^	0.56^c^	0.52^b^	0.59^c^	0.57^c^
**Bodyfat distribution**						
BMI SDS	0.95^c^	0.75^c^	0.71^c^	0.49^b^	0.58^b^	0.58^c^
SAD	0.94^c^	0.72^c^	0.66^c^	0.49^b^	0.65^c^	0.58^c^
SAD/height	0.86^c^	0.82^c^	0.71^c^	0.62^c^	0.62^c^	0.56^c^
Waist circ.	0.97^c^	0.68^c^	0.63^c^	0.43^a^	0.63^c^	0.64^c^
Waist circ./height	0.92^c^	0.72^c^	0.71^c^	0.54^b^	0.53^c^	0.63^c^
Upper thigh circ.	0.93^c^	0.58^c^	0.60^c^	0.36^a^	0.64^c^	0.55^c^
Neck circ.	0.84^c^	0.56^c^	0.48^b^	0.23	0.63^c^	0.47^b^
Upper arm circ.	0.96^c^	0.72^c^	0.75^c^	0.56^b^	0.64^c^	0.45^a^
**Skinfold measurements**						
Biceps	0.81^c^	0.82^c^	0.71^c^	0.50^b^	0.55^c^	0.66^c^
Triceps	0.83^c^	0.79^c^	0.67^c^	0.52^b^	0.50^b^	0.65^c^
Subscapular	0.87^c^	0.80^c^	0.67^c^	0.50^b^	0.63^c^	0.58^c^
Supra-iliac	0.87^c^	0.84^c^	0.71^c^	0.57^c^	0.61^c^	0.60^c^

^a^ *P* < 0.05, ^b^ *P* < 0.01, ^c^ *P* < 0.001.

Abbreviations as in Table 1 and *r*_s_ represents Spearman rank correlation coefficient.

All glycemic parameters (except plasma glucose) were significantly correlated with all body fat characteristics, including skinfolds and many of the lipid and apolipoproteins tested. Among the glycemic parameters analyzed, c-peptide showed the strongest correlations with plasma lipids, apolipoproteins, body fat distribution parameters, and skinfold measurements (not shown). For associations, lacking statistical significance, see Supplemental Table 2.

### Blood pressure

In general, SBP was more strongly correlated than DBP with most variables studied. SBP correlated significantly with hs-CRP, fasting insulin, proinsulin, and c-peptide and with all the variables for body fat distribution, including skinfold measurements (Table 2). In adolescents with obesity, SBP correlated to RA-Med thickness (*r*_s_ = 0.52; 0.049, not shown).

### Receiver operating characteristic curve analysis

ROC analysis yielded high *c*-values for RA I/M (0.86), and for hs-CRP (0.88), revealing the strength in indicating the correct study group to which the individual belonged. Thus, the individual values for RA I/M and hs-CRP strongly indicated which study group the adolescent belonged to, obese or non-obese, whereas RA IMT was of no use (Supplemental Figure 3). The *c*-value for RA intima thickness was 0.71 (not shown).

## Discussion

In this study, obese adolescents showed more signs of vascular aging in the RA than their normal-weight peers. The hs-CRP results increased exponentially with increasing adiposity and correlated significantly with RA I/M results, indicating a link between adiposity, inflammation, and vascular aging in these adolescents, further supported by the results of the ROC analysis. A link between adolescent adiposity – inflammation and adverse vascular effects has been reported previously (6).

In accordance with previous reports, serum lipid and apolipoprotein concentrations were significantly higher in the obese adolescents. Most of these correlated significantly with the hs-CRP results, but the correlation with RA I/M was only weak and not significant, which could indicate that the atherosclerotic process in these adolescents was more affected by inflammation than by dyslipidemia. We recently found that levels of the inflammation marker pentraxin3 were significantly positively correlated with the intima thickness and I/M and negatively correlated with the media thickness of the CCA in a mixed group of women with normal or preeclamptic pregnancies (18).

The wall dimensions of both arteries were significantly correlated with many of the analyzed body fat distribution and laboratory variables, but the correlations were generally stronger for the dimensions of the muscular RA than for those of the elastic CCA, and significant group differences were found only for the RA measurements. In a systemic review, studies using the CIMT, correlations with CVD risk factors, and body fat characteristics are usually weaker (19) than our estimated correlations based on the RA I/M. Furthermore, correlations between the CCA wall layer values and those of the RA were weak, supporting our finding that adiposity in adolescents might more adversely affect the muscular RA than the elastic CCA. The muscular RA, having similar histological structure and dimensions to those of the coronary arteries (20), might better reflect the adverse effects of adiposity than the elastic CCA. Alternatively, the lack of significant group differences for the CCA dimensions might also be due to the limited sample size and effects of a type two error.

In some studies, included in a systemic review, the CIMT was not significantly increased in obese adolescents (19), in accordance with our findings. However, previous studies that did report increased CIMT in obese adolescents used lower probe frequencies (e.g. 7–12 MHz). A recent review of studies using probe frequencies of 7.5–12 MHz found that the CIMT was thicker in obese/overweight children and adolescents whether they were metabolically healthy or unhealthy, compared with metabolically healthy normal-weight children and adolescents (21). When low-frequency probes are used, the CIMT estimate includes the thickness of the peripheral adventitia layer (22). Inflammation increases both the arterial intima and the adventitia layers (23), contributing to an early increase in CIMT, before the subsequent reduction in media thickness (13, 14) affects estimates of the CIMT in the reverse direction. In support, the RA I/M, but not RA-IMT, was strongly correlated with most body fat distribution parameters, skinfold measurements, except neck circumference, as well as with several glycemic parameters and yielded excellent *c*-values indicating group belonging.

Visceral fat is considered more harmful to cardiovascular health than subcutaneous fat. In elderly, both waist/height and SAD/height ratio strongly predict visceral fat as assessed by dual-energy x-ray absorptiometry (DEXA), yielding excellent *c*-values in ROC analysis; 0.90 and >0.83, respectively (24). In fact, among the anthropometric measures we studied, the SAD/height and waist circumference/height correlated most strongly with vascular aging, even stronger than the waist circumference otherwise suggested as a routine vital sign in clinical practice (25). Furthermore, the significant group difference in radial I/M ratio remained after adjustments including BMI, but not after adjustment including SAD/height. Thus, our results are in line with results in a recent report on obesity in adolescents, in which visceral fat as assessed by DEXA was significantly correlated with the cardiovascular risk marker pulse wave velocity (PWV) (26), both reports supporting that visceral fat mass is more harmful to cardiovascular health than subcutaneous fat.

Increased weight and adiposity are associated with increased serum levels of certain vascular inflammation markers, impaired microvascular endothelial function (27), and increased PWV, the latter normalized in adolescents regaining normal fat mass (28). Thickening of the arterial intima layer is an early morphological sign of atherosclerosis (5) and is associated with aging (13, 29), child obesity (30, 31), hypertension (32), and coronary heart disease (33). In a Swedish cohort of 70-year-olds, the CCA intima was significantly thicker among those with prevalent CVD, myocardial infarction, stroke, hypertension, or hyperlipidemia, and the intima thickness was significantly associated with higher BMI, waist circumference, years of hypertension, years of hyperlipidemia, and number of cigarettes smoked/week (14). Recent reports confirmed that intima thickness is a more accurate marker of atherosclerosis than IMT (34), and that intima thickness in different arteries, more strongly than IMT, could identify patients with ischemic stroke (35). However, in a report, many years ago, I/M ratio was the best dimension for estimating the degree of atherosclerosis from the patient’s histology (36). Since many years, we use both the intima and I/M thickness ratio because together they better reflect the differential changes occurring during the atherosclerotic process; the initial increase in intima thickness (5) and the subsequent reduction in media thickness resulting in an amplified increase in the I/M ratio, which better reflects e.g. the effects of aging and/or the duration of the condition under study (12, 13). We have repeatedly found logical group differences and associations when using this principle, in contrast to the lack of correlations when using IMT (11–14, 16, 18, 37–40).

### Strengths and limitations

Our data add information about the negative effects of adolescent adiposity on vascular aging in the muscular RA – which has a similar histological structure and dimensions to those of the coronary arteries. The study sample was limited, but findings for the RA were consistent, with highly significant study group differences, logical, strong, and consistent associations with cardiovascular risk factors, and excellent *c*-values in the ROC analysis, indicating the study group to which the participants belonged. The limited sample size and risk of type II error could have contributed to the non-significant findings for CCA; alternatively, the muscular RA might better reflect adverse vascular effects of adolescent adiposity than the elastic CCA. The overwhelming majority of participants in both study groups were Caucasians. Only for a few in each study group, the ethnicity could not be found.

### Summary

Adolescents with obesity had significantly more signs of vascular aging in the muscular RA, than normal weight peers. The RA I/M correlated significantly with hs-CRP levels, and both correlated logically with the analyzed body characteristics, serum lipid fractions, and glycemic parameters. Both the RA I/M and hs-CRP levels yielded excellent *c*-values in the ROC analysis, indicating the study group to which the participants belonged: obese or non-obese. The seemingly exponential increase in hs-CRP levels with increasing obesity, the generally stronger correlations of cardiovascular risk factors with hs-CRP levels than with the RA I/M, and the excellent *c*-values in the ROC analysis support a link between adiposity and inflammation in the development of vascular aging in these adolescents.

## List of Abbreviations

**Table:** 

BMI	body mass index
CCA	common carotid artery
CIMT	carotid intima-media thickness
CRP	C-reactive protein
CVD	cardiovascular disease
DEXA	dual-energy x-ray absorptiometry
hs-CRP	high-sensitivity CRP
I/M	intima/media thickness ratio
IMT	intima-media thickness
PWV	pulse wave velocity
RA	distal radial artery
ROC	receiver operating curve
r_s_	Spearman rank correlation coefficient
SAD	sagittal abdominal diameter
s-BMI	standardized (age- and gender-adjusted) BMI
type II error	the risk of not detecting a true difference due to small numbers
UHRU	ultra-high-resolution ultrasound

## Supplementary Material

Obesity in adolescents associated with vascular aging – a study using ultra-high-resolution ultrasound
