# Supplementary material for: Obesity in adolescents associated with vascular aging – a study using ultra-high-resolution ultrasound
Source: Ups J Med Sci. 2022 Jun 3;127:10.48101/ujms.v127.8676. doi: 10.48101/ujms.v127.8676 (PMC9254329; doi:10.48101/ujms.v127.8676)

Supplemental Table 1. Clinical characteristics by study group, lacking statistically significant group differences.

**Variable**	**Obese adolescents (n = 19)**	**Normal-weight adolescents (n = 20)**
	Median (IQR)	Median (IQR)
Age (y)	14 (12, 15)	14 (12, 15)
Women/Men	7/12	7/13
Height (cm)	164 (155, 177)	164 (160, 174)
DBP (mmHg)	71 (66, 75)	66 (63, 74)
**Common carotid artery (N=39)**		
Intima thickness (mm)	0.07 (0.07, 0.08)	0.075 (0.07, 0.08)
Media thickness (mm)	0.49 (0.43, 0.58)	0.52 (0.43, 0.60)
Intima/Media ratio	0.16 (0.13, 0.17)	0.15 (0.13, 0.18)
CIMT	0.57 (0.50, 0.66)	0.60 (0.50, 0.68)
**Radial artery (N = 32)**	(n = 15)	(n = 17)
Media thickness (mm)	.086 (0.072, 0.101)	0.098 (0.089, 0.123)
RA-IMT	0.112 (0.100, 0.129)	0.123 (0.115, 0.148)

**Plasma lipids**		
Total cholesterol (mmol/L)	4.1 (3.8, 4.7)	4.1 (3.5, 4.3)
**Glycaemic parameters**		
Fating Glucose	5.6 (5.4, 5.9)	5.8 (5.5, 6.1)

Data are presented as medians (25% and 75% IQR) or numbers.

The Wilcoxon sum rank test was used to test for group differences.

Abbreviations, as in Table 1 and CIMT, Carotid intima-media-thickness;

RA, radial artery; IMT, intima-media thickness

Supplemental Table 2. Associations within the combined study groups of adolescents with or without obesity, not reaching statistical significance.

Variable	BMI	f-Insulin	Hs-CRP	RA-I/M ratio	Systolic BP	LDL/HDL
	r_s	r_s	r_s	r_s	r_s	r_s
**Common carotid artery**						
Intima thickness	-0.09	0.01	-0.15	0.25	0.02	-0.29
Media thickness	-0.05	0.04	-0.26	0.09	-0.09	-0.01
Intima/Media ratio	0.00	-0.03	0.13	0.02	0.08	-0.15
CIMT	-0.06	0.04	-0.26	0.09	-0.09	-0.2
**Radial artery (N = 32)**						
RA-IMT	0.06	-0.12	-0.08	-0.32	0.23	-0.31
**Glycaemic parameters**						
f-Glucose	-0.06	0.28	-0.12	0.25	-0.05	-0.08

Abbreviations, as in Table 2.

Supplemental Figure 1. Ultra-high resolution ultrasound (50 MHz) of a distal radial artery far wall.

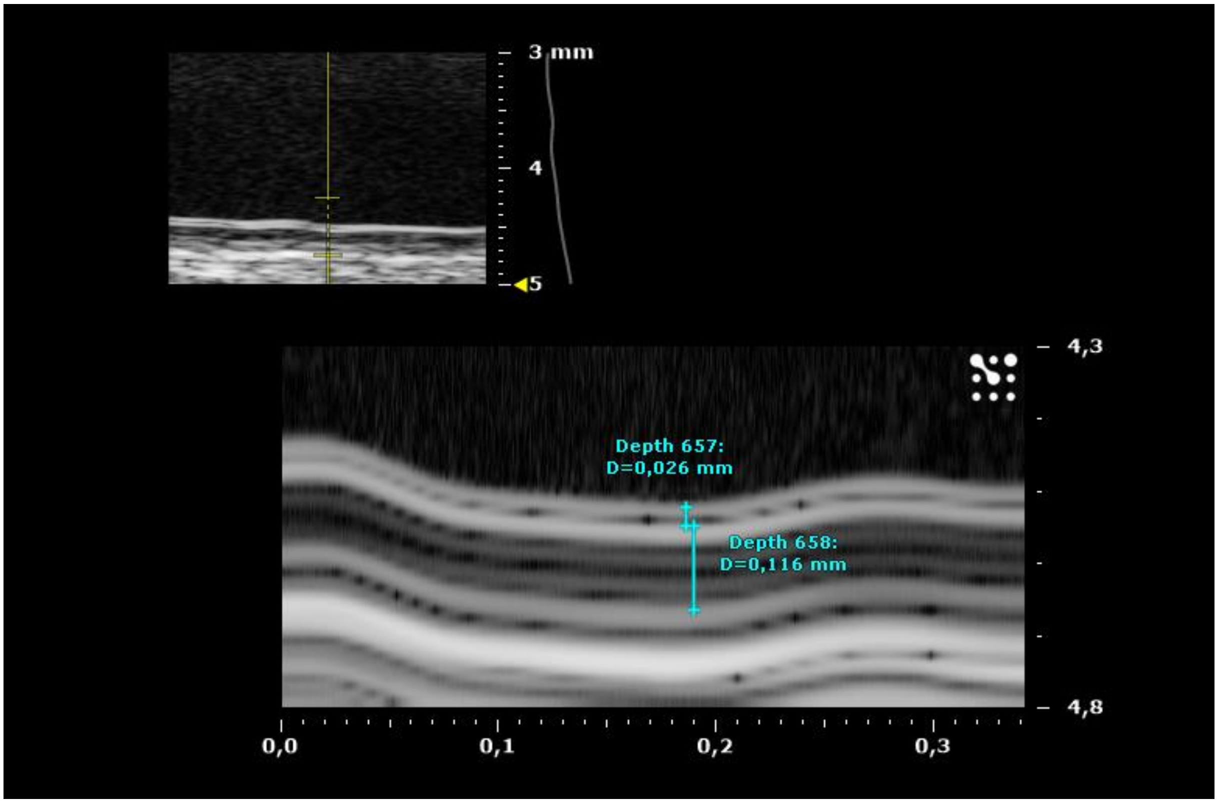

Radial intima and media thicknesses indicated.

Supplemental Figure 2. Correlation of plasma hs-CRP levels with **(A)** triceps skinfold measurements and **(B)** waist circumference/height ratios. **(C)** Correlation of sagittal abdominal diameter (SAD)/height with radial artery intima/media thickness ratio (I/M).

A

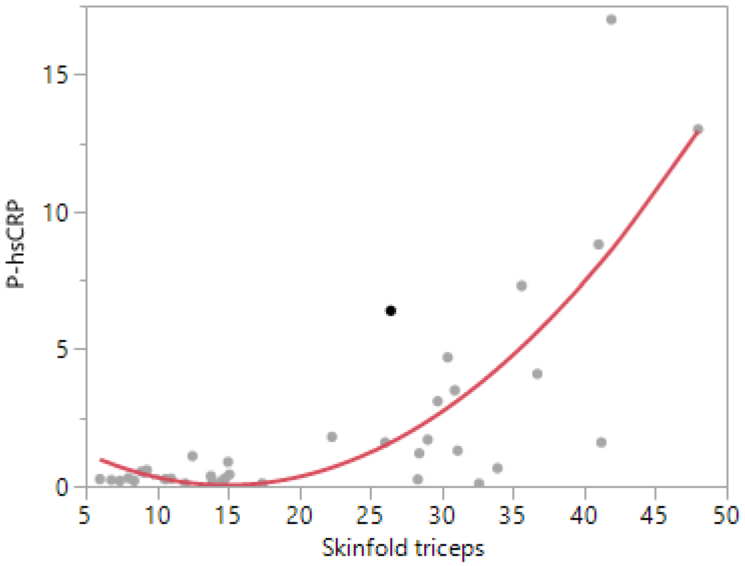

Spearman rank correlation coefficient, (r_s) = 0.67; <0.001)

Quadratic fit; t ratio = 3.87; $p < 0.001$

B

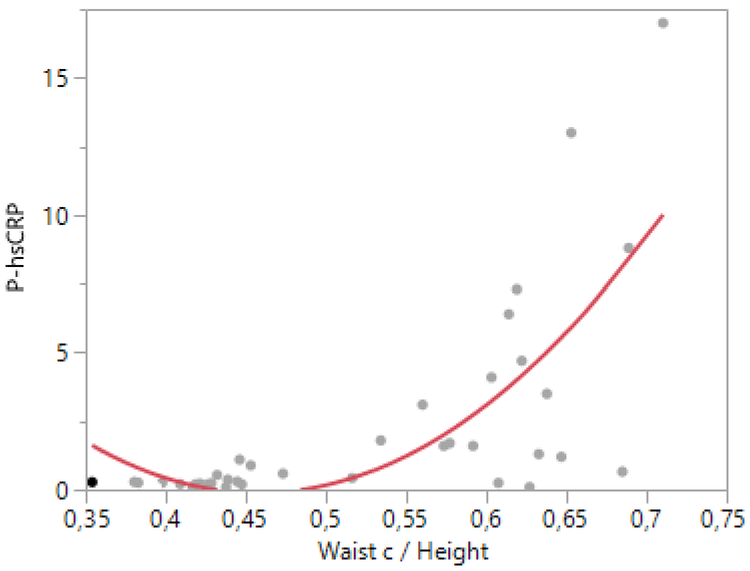

$r_s = 0.71$; $p < 0.001$; Qudratic fit; t ratio = 2.84; $p < 0.01$

C

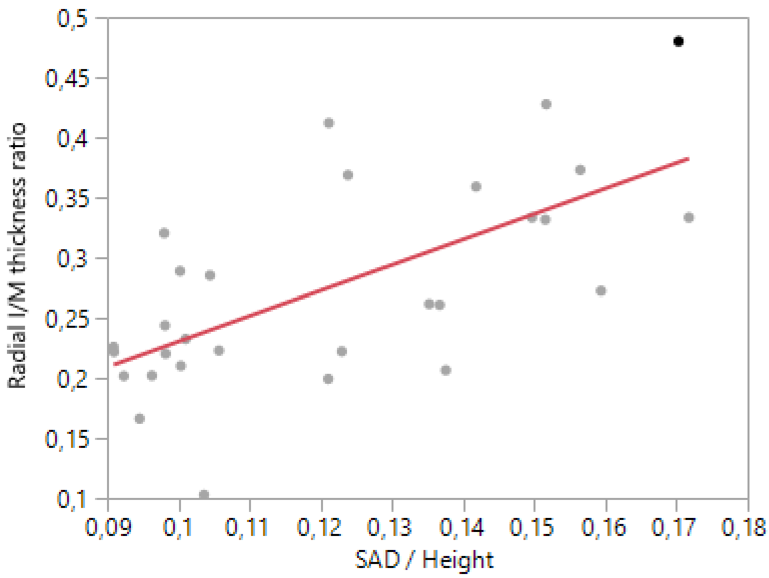

$r_s = 0.62$; <0.001

Supplemental Figure 3. Receiver operating characteristic (ROC) curve analysis based on radial artery intima/media thickness ratio (I_M ratio), intima-media-thickness (IMT) and plasma hs-CRP measurements, indicating the ability to correctly indicate assignment of adolescents to obese or non-obese groups.

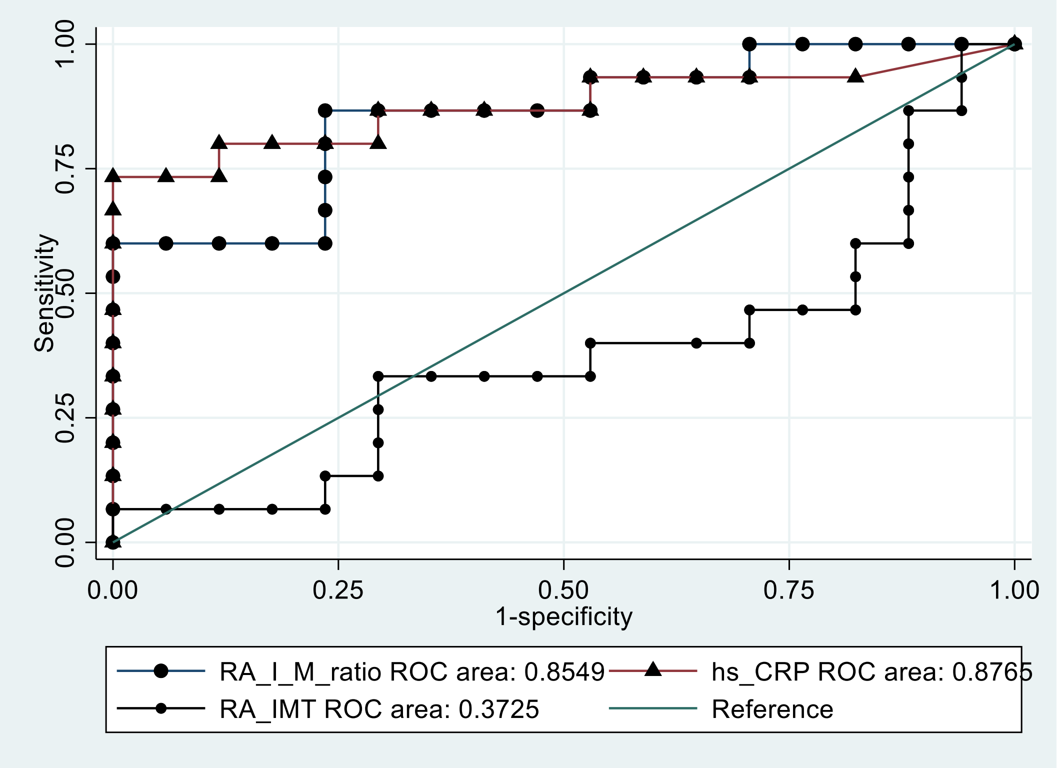

Supplement: Obesity in adolescents associated with vascular aging – a study using ultra-high-resolution ultrasound [file UJMS-127-8676-s001.pdf]
